# Recent Food Shortage Is Associated with Leprosy Disease in Bangladesh: A Case-Control Study

**DOI:** 10.1371/journal.pntd.0001029

**Published:** 2011-05-10

**Authors:** Sabiena G. Feenstra, Quamrun Nahar, David Pahan, Linda Oskam, Jan Hendrik Richardus

**Affiliations:** 1 Department of Public Health, Erasmus MC, University Medical Center Rotterdam, Rotterdam, The Netherlands; 2 Health Systems and Infectious Diseases Division, ICDDR, B, Dhaka, Bangladesh; 3 Rural Health Program Nilphamari, The Leprosy Mission Bangladesh, Dhaka, Bangladesh; 4 KIT Biomedical Research, Amsterdam, The Netherlands; Kwame Nkrumah University of Science and Technology (KNUST) School of Medical Sciences, Ghana

## Abstract

**Background:**

Leprosy is remaining prevalent in the poorest areas of the world. Intensive control programmes with multidrug therapy (MDT) reduced the number of registered cases in these areas, but transmission of *Mycobacterium leprae* continues in most endemic countries. Socio-economic circumstances are considered to be a major determinant, but uncertainty exists regarding the association between leprosy and poverty. We assessed the association between different socio-economic factors and the risk of acquiring clinical signs of leprosy.

**Methods and Findings:**

We performed a case-control study in two leprosy endemic districts in northwest Bangladesh. Using interviews with structured questionnaires we compared the socio-economic circumstances of recently diagnosed leprosy patients with a control population from a random cluster sample in the same area. Logistic regression was used to compare cases and controls for their wealth score as calculated with an asset index and other socio-economic factors. The study included 90 patients and 199 controls. A recent period of food shortage and not poverty *per se* was identified as the only socio-economic factor significantly associated with clinical manifestation of leprosy disease (OR 1.79 (1.06–3.02); p = 0.030). A decreasing trend in leprosy prevalence with an increasing socio-economic status as measured with an asset index is apparent, but not statistically significant (test for a trend: OR 0.85 (0.71–1.02); p = 0.083).

**Conclusions:**

Recent food shortage is an important poverty related predictor for the clinical manifestation of leprosy disease. Food shortage is seasonal and poverty related in northwest Bangladesh. Targeted nutritional support for high risk groups should be included in leprosy control programmes in endemic areas to reduce risk of disease.

## Author Summary

Although intensive control programs reduced the prevalence of leprosy worldwide, new cases of this infectious disease are still detected in several of the poorest areas of the world. Therefore the disease is known as a disease of poverty. To be able to control the disease it is important to know which aspects of poverty play a role in transmission and acquiring clinical signs of disease. In this study socio-economic circumstances of recently diagnosed leprosy patients were compared with those of a control population in the poverty stricken northwest area of Bangladesh where leprosy is common. A recent period of food shortage was the only socio-economic factor that was found related to leprosy disease in this study and not poverty as such. Food shortage is seasonal and poverty related in northwest Bangladesh, while malnutrition is known to lower immunity and make people more vulnerable to infectious diseases. Therefore it was concluded that malnutrition as an aspect of poverty played an important role in the development of the clinical signs of leprosy. We therefore recommend that nutritional support for high risk groups should be included in leprosy control programmes to reduce risk of disease in areas where leprosy is common.

## Introduction

Leprosy is known as a disease of poverty. Only in the poorest areas of the world the infectious disease caused by *Mycobacterium leprae* is still endemic. A causal relationship between poverty and leprosy is difficult to demonstrate, and uncertainty exists about how leprosy and poverty are associated [1],[2].

Bangladesh is one of the countries where the disease is still endemic. Despite reaching the ‘elimination’ target of less than one registered case per 10,000 inhabitants for the whole country in 1998, the prevalence is still above target in some of the poorest areas of Bangladesh [3],[4]. In the poverty stricken northwest part of the country, where The Leprosy Mission Bangladesh is operating a leprosy control programme, the new case detection rate was still 1.25 per 10,000 inhabitants in 2008.

To generate more knowledge about risk factors for leprosy and to assess the effect of new interventions, a research project was initiated in northwest Bangladesh in 2001: the COLEP study, a prospective (sero-) epidemiological study on contact transmission and chemoprophylaxis in leprosy [5]. The first results of the study indicated that prophylactic treatment with rifampicin is a promising way to prevent leprosy in contacts of patients [6]. Physical distance to a patient and the severity of the disease (leprosy classification) were identified as risk factors associated with transmission of *Mycobacterium leprae* to contacts of a patient. Furthermore, the host characteristics “blood relationship to the patient” and “age” were identified as risk factors for the development of clinically apparent disease, while a previous vaccination with BCG had a preventive effect [7]. These findings indicate that innate and acquired immunity affects the development of clinical signs of leprosy. Clinical disease occurs most probably in only 1–5% of persons infected with *Mycobacterium leprae*, after an incubation period of several years.

The objective of this study, which is part of the COLEP project, was to assess the association between poverty and leprosy more closely, by measuring the effects of different socio-economic factors on acquiring clinical signs of leprosy disease.

## Methods

### Study area and population

A case-control study was carried out in August 2009 in the districts of Nilphamari and Rangpur in northwest Bangladesh. This large (3951 km^2^) - mainly rural - area has app. 4.5 million inhabitants and is one of the poorest parts of Bangladesh [8],[9].

The first 110 new leprosy patients registered in 2009 in the study area were selected as cases. These patients were diagnosed by The Leprosy Mission Bangladesh or government facilities according to the national guidelines [10]. Only one patient per household was interviewed to avoid bias due to clustering. From the initially selected group, 10 people could not been reached, while one was excluded because he was living in the same household as another selected patient.

Controls without leprosy were randomly selected from a referent group, representative for the general population in the area. This group was selected at the start of the COLEP study in 2002 by a multi-cluster sampling procedure [11]. Twenty clusters of 1000 people each were randomly selected from the 13 sub-districts in this area. In each of the sub-districts one to three clusters were allocated proportional to the population size. Within the sub-districts first unions and thereafter sub-unions were selected randomly by computerized sampling. In each of the thus created clusters, everyone willing to participate and available on the day of registration was included. Registration started at the northern border of the selected village or urban ward and continued until 1000 people were included in the cluster.

For this study, 15 people were randomly selected from each of the 20 clusters by computerized sampling. The 15 selected candidates of each cluster were numbered one to fifteen. Interviewers started to contact the first person and continued following the numbering until 10 people were interviewed or everyone was contacted. Controls were excluded when they were ever diagnosed as leprosy patient or if they came from the same household as another participant in the study.

### Data collection

Research staff of The Leprosy Mission Bangladesh carried out home visits to conduct interviews with pre-tested structured questionnaires. Besides questions on personal data and some details about their disease (for patients only), participants were asked about their living circumstances and economic situation. They were asked about ownership of assets, including housing, drink water supply, sanitary facilities, livestock and land, while they were also questioned about educational level, job status, monthly household income, seasonal income variations, changes in economic and living situation due to the disease leprosy as well as over the last three years in general, and periods of food shortage in the previous year and ever in life. Food shortage was defined as a period in which a family had to reduce the number of meals a day or had to reduce the intake of foods other than rice, like vegetables, fruits, meat or fish.

### Analysis

Data from the questionnaires were entered into an Access database. After data cleaning, analysis was performed using the statistical package STATA version 10.0.

Socio-economic status of the participants was estimated by an asset index. Factor analysis, principal components factor, as described by Filmer and Pritchett was used to construct an asset index to assign a wealth score to all participants [12]. Data on ownership of different assets in their household was used to calculate a wealth score by weighing the response for each asset of their household by the coefficient of the first factor as determined by application of the factor analysis, and summing the results (Table 1). Data regarding possession of a car, rickshaw, animal cart, and drink-water supply were not correlated with the wealth scores as calculated and therefore excluded from the final model. The control group was assigned to five wealth quintiles according to their final score. Cases were assigned to these quintiles according to the threshold values set by the control group.

Logistic regression was used to compare cases and controls for the wealth score quintile and the other factors measuring aspects of socio-economic situation: income level, educational level of the highest educated person in the household, household size, crowding (defined for this study as more than three people per sleeping room on average), food shortage ever and a period of food shortage in the last year. Univariate and multivariate logistic regression with a backwards elimination procedure was used to assess the association between these factors as well as the potential confounding factors age and sex.

**Table 1:** Variables in the asset index with weighing value as obtained by factor analysis (first factor).

Description of assets	Number and % possessing the asset	Weighing value in the final formula
Floor of house: earth, mud or clay	254 (87.9%)	−0.5990
Floor of house: bamboo or wood	10 (3.5%)	0.1100
Floor of house: cement, tiles or carpet	25 (8.7%)	0.6237
Roof of house: bamboo, thatch	22 (7.6%)	−0.2038
Roof of house: tin	267 (92.4%)	0.2038
Walls of house: mud, bamboo or palm	209 (72.3%)	−0.5467
Walls of house: tin	39 (13.5%)	0.1328
Walls of house: cement or bricks	41 (14.2%)	0.5710
Electricity	102 (35.3%)	0.6874
Radio	36 (12.5%)	0.1877
Television	81 (28.0%)	0.7294
Computer	5 (1.7%)	0.2094
Mobile phone	110 (38.1%)	0.6272
Refrigerator	6 (2.1%)	0.1696
Fan	87 (30.1%)	0.7295
Air conditioner	3 (1.0%)	0.1586
Almirah or wardrobe	126 (43.6%)	0.6567
Table	240 (83.0%)	0.5185
Chair	210 (72.7%)	0.6167
Watch or clock	167 (57.8%)	0.6183
Bicycle	133 (46.0%)	0.5943
Van or rickshaw	31 (10.7%)	x
Animal drawn cart	10 (3.5%)	x
Motorcycle or scooter	11 (3.8%)	0.4073
Tractor or motorized farm equipment	29 (10.0%)	0.2934
Local rice husking equipment	77 (26.6%)	0.2661
Car or truck	2 (0.7%)	x
Owns livestock	251 (86.9%)	0.2189
Owns the house	281 (97.2%)	0.1879
Owns the land of the house	253 (87.5%)	0.4170
Owns farmland	164 (56.8%)	0.4187
Drink water from tube well/bore hole	280 (96.9%)	x
Flush toilet or septic tank	3 (1.0%)	0.2606
Latrine	222 (76.8%)	0.3632
No toilet facility (bush/field)	64 (22.2%)	−0.4327
Shares toilet	48 (16.6%)	−0.1281
**Total number of participants**	**289**	

### Ethics statement

All participants received verbal information about the study and were asked to sign a consent form. Ethical approval for this study was obtained from the Bangladesh Medical Research Council (under reference: BMRC/NREC/2007-2010/2107).

## Results

Initially 99 patients (cases) and 199 controls were included in the study population. A deterioration of socio-economic or living condition due to the disease was mentioned by 9 (8.9%) of the cases. All these patients had severe forms of leprosy; 6 had grade II disabilities, while the other 3 had the more severe MB form of leprosy. Because the objective of this study was to assess the socio-economic condition as a risk factor for developing clinical signs of leprosy disease, it was important to establish the situation around the time the disease became apparent. We therefore excluded for further analysis the 9 cases in which the economic situation had changed due to the disease, to avoid confusion about cause and effect.

Of the 90 patients included for analysis, the sex ratio (M/F) of the was 1.2; 21.1% had the multibacillary (MB) form of the disease, while 6.6% was diagnosed with a grade II disability, according to the WHO classification (Table 2). The child rate (<15 years of age) was 15.6%. At the time of the interview, 58.9% of the cases were still on multidrug therapy (MDT), while the other 41.1% had just completed their therapy and were released from treatment.

**Table 2:** General characteristics of the leprosy cases in the analysis population.

	Male			Female			
Age group (in years)	Case N (%)	MB (% of cases)	Disability grade II (% of cases)	Case N (%)	MB (% of cases)	Disability grade II (% of cases)	Total N (%)

Both the case and control populations were distributed randomly throughout the study area. The control group was representative for the general population in the area with respect to the household characteristics religion, household composition, educational level, and living area (urban/rural), as compared to the national statistics, but males in the working age (20–39 years) were slightly underrepresented in the control group [8],[9].

The prevalence of leprosy decreased with an increased level of economic status, measured by the wealth score quintile (test for a trend: OR 0.85 (0.71–1.02); p = 0·083, Table 3). Uni- and multivariate logistic regression analysis revealed only a statistically significant association of the socio-economic factor “a self reported period of food shortage in the last year” with leprosy disease (OR 1.79 (1.06–3.02); p = 0.030, Table 3). None of the other socio-economic factors were associated with leprosy disease.

**Table 3:** Results of univariate and multivariate logistic regression analysis with a backwards elimination procedure.

						Univariate			Multivariate		
Variable		Control N (%)		Case N (%)		Crude Odds Ratio (95% CI)		p-value	Odds ratio (95% CI)		p-value
**Wealth quintile (asset index)**	1	40	(20.1%)	25	(27.8%)	1.00					
	2	40	(20.1%)	20	(22.2%)	0.80	(0.38–1.67)				
	3	40	(20.1%)	16	(17.8%)	0.64	(0.30–1.38)				
	4	40	(20.1%)	17	(18.9%)	0.68	(0.32–1.45)				
	5	39	(19.6%)	12	(13.3%)	0.49	(0.22–1.12)				
	Assuming a linear trend					0.85	(0.71–1.02)	p = 0.083			
**Income level (BDT)**	Mean	4108		4853							
	Std. Dev.	3978		3991		1.00	(1.00–1.00)	p = 0.148			
Educational level*	High	113	(56.8%)	49	(54.4%)	1.00					
	Low	86	(43.2%)	41	(45.6%)	1.10	(0.67–1.81)	p = 0.711			
**Household size**	Mean	5.28		4.96							
	Std. Dev.	2.30		1.97		0.93	(0.82–1.05)	p = 0.248			
**Crowding** ‡	No	129	(64.8%)	55	(61.1%)	1.00					
	Yes	70	(35.2%)	35	(38.9%)	1.17	(0.70–1.96)	p = 0.544			
**Ever food shortage**	No	76	(38.2%)	30	(33.3%)	1.00					
	Yes	123	(61.8%)	60	(66.7%)	1.24	(0.73–2.09)	p = 0.428			
**Food shortage in the last year**	No	128	(64.3%)	47	(52.2%)	1.00			1.00		
	Yes	71	(35.7%)	43	(47.8%)	1.65	(1.00–2.74)	p = 0.052	1.79	(1.06–3.02)	p = 0.030
**Sex**	Female	116	(58.3%)	41	(45.6%)	1.00					
	Male	83	(41.7%)	49	(54.4%)	1.67	(1.01–2.76)	p = 0.045			
**Age (years)**	<10	22	(11.1%)	6	(6.7%)	1.00			1.00		
	10–19	65	(32.7%)	20	(22.2%)	1.13	(0.42–3.17)	p = 0.819	1.17	(0.41–3.32)	p = 0.762
	20–29	27	(13.6%)	21	(23.3%)	2.85	(0.98–8.30)	p = 0.054	3.22	(1.09–9.51)	p = 0.034
	30–39	32	(16.1%)	15	(16.7%)	1.72	(0.58–5.12)	p = 0.331	1.84	(0.61–5.55)	p = 0.277
	40–49	35	(17.6%)	11	(12.2%)	1.15	0.37–3.56)	p = 0.805	1.28	(0.38–3.67)	p = 0.781
	50+	18	(9.1%)	17	(18.9%)	3.46	(1.13–10.61)	p = 0.030	3.56	(1.15–11.02)	p = 0.028
	**Total**	**199**	**(100%)**	**90**	**(100%)**						

*Educational level: Low: highest educated person in the household had 0–5 years of schooling; High: highest educated person had more than 5 years of schooling.

‡Crowding: for this study defined as more than three people per sleeping room (average).

## Discussion

A recent period of food shortage and not poverty *per se* was identified as the only socio-economic risk factor significantly associated with clinical manifestation of leprosy disease in northwest Bangladesh. A decreasing trend in leprosy prevalence with an increasing socio-economic status as measured with an asset index is apparent, but not statistically significant.

The strength of this case control study is that it takes into account recently diagnosed leprosy cases, while patients who reported changes in economic or living situation due to their disease were excluded. In this way the actual situation at the time of diagnosis could be measured, making it possible to draw conclusions about the association of leprosy and socio-economic situation as risk factor for acquiring clinical signs of leprosy disease.

A limitation of the study is the use of self-reported data on income, educational level and food shortage as measured by a questionnaire, which is by definition subjective. The effect of this form of bias was reduced by asking cases and controls the same questions. Furthermore also an asset index as proxy to measure wealth was constructed, which is a more objective measure for socio-economic status of the household.

Although objective, a limitation of the use of a wealth index is that the score of the index depends highly on the set of assets used [13]. Since the asset index used in the USAID sponsored Demographic and Health Survey, carried out in 84 developing countries, has a proven valuable for public health purposes we used a set of assets based the local version of the Demographic and health Survey for Bangladesh [14], [15]. Another limitation of this method is that the index is relative and based on the assets of others in the group. The whole assessed group is divided into five equal quintiles based on their wealth score. Since the majority of people are very poor in the study area in northwest Bangladesh, people assigned to the higher quintiles have more assets and are somewhat better off than the households included in lower quintiles, but can not be considered as rich by any means in this poverty stricken area.

It is likely that most people who reported “food shortage in the last year” in our study observed shortage of food in the yearly period of seasonal income shortage in rural Bangladesh which lasts from the end of September until November, just after the rainy season and before the main rice harvest in November/December. In this period there are few work opportunities, low household food stocks, and increased rice prices. The yearly period of food shortage roughly coincides with the start of symptoms of leprosy in the selected cases, as 70% of the patients reported start of their symptoms less than six months before they were registered (between seven to twelve months before the interview, between September to December 2008).

In poor rural communities in Bangladesh seasonal income changes are common. In our study the reported income changed from a monthly average of 3000 BDT (43 US$) to 9000 BDT (130 US$) per household. Seasonal income changes are closely related to daily expenditure on food and influences the nutritional status of the people in rural Bangladesh [16]. In rural Bangladesh, chronic energy deficiency (CED) based on body mass index (BMI) is high (between 60–70%) in all age and sex groups, while seasonal differences in energy intake are substantial in all age and sex group as well [17]. The amount of rice consumed is quite stable, but expenditure on high nutritious and more expensive food decreases in months of low income in rural communities, likely causing micronutrient deficiencies. Studies in Bangladesh revealed an association between the proportion of expenditure on non-rice food and maternal underweight as well as child stunting [18], and an association between a low BMI and increased mortality in adults [19].

**Figure 1:**
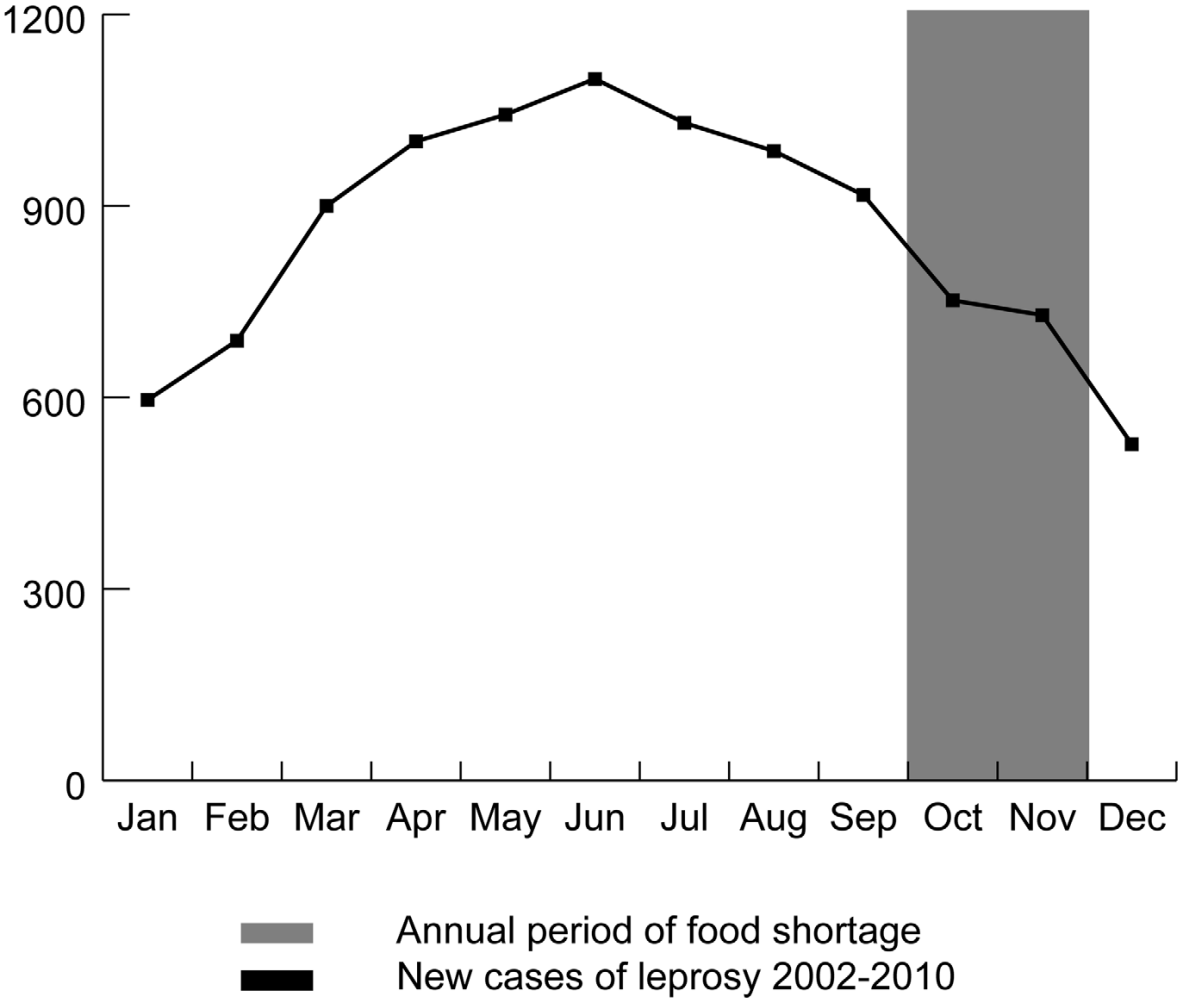
Seasonal pattern of leprosy cases in the study area (2002–2010) in relation to the annual period of food shortage.

The hypothesis that seasonal food deficiencies might be associated with leprosy is strengthened by the seasonal pattern in number of new leprosy cases registered per month over the last nine years (2002–2010) in the districts where the study was carried out. The number of newly registered cases is rising from February, about four months after the start of the seasonal low-income period, and reaches a maximum in June at the beginning of the monsoon period in Bangladesh and six months after the end of the low-income period (figure 1).

However alternative explanations are possible. A study in a leprosy endemic area in India showed a strong seasonal pattern in *Mycobacterium leprae* bacteria detectable in the general population by nasal PCR and salivary ML-IgA positivity. The rates of PCR positive nasal swabs were high in the period immediately after the monsoon rains from July to November, while salivary ML-IgA titres were high in November at the end of the wet period. This indicates a seasonal pattern in exposure to *Mycobacterium leprae* [20].

“Food shortage in the last year” as assessed in this study represents a recent (short) period of poverty with limited expenditure on high nutritious food, likely causing nutritional deficit. In contrast, an asset index as a proxy to measure wealth gives an indication of the long-term economic status of a household, since people tend not to sell their assets in seasonal short periods of low income, but only in longer term poverty [12],[21].

Although the general population sample (referent group) of the COLEP trial was selected almost seven years before this study, a selection of this group is still suitable to use as control group. Only three of the selected leprosy cases were born less than seven years before the start of the study, from which you can conclude that leprosy below this age is rare. Furthermore 80% of the selected people of the control group participated in the study, which indicates that the population in this area is not very mobile. However, due to the original selection method used for this referent group, men in the working age are underrepresented, since many of them were absent from their house at the time of registration. Therefore age and sex were included as potential confounders in the analysis.

The actual association between poverty and leprosy might be stronger than indicated by this study, because only registered cases were included in the study. Registered cases receive leprosy treatment and have access to health services. Although the area has a long running active disease control programme in which treatment is given free of charge, there are still people who have no access to these services. In a study carried out in 2002 in northwest Bangladesh, the population prevalence of leprosy was found to be six times higher than the registered prevalence [11]. The fact that 11% of the original selected cases in our study had grade II disability, indicating late detection of the disease, suggests that there may be undetected leprosy cases in the area. Poverty is one of the reasons for limited access to leprosy care. Stigma, although less common due to the active control and health education activities in the area, and cultural defined limited access to health care for women might be of importance as well [22].

An association between food shortage and leprosy was also observed in Brazil [1]. However, in Brazil a period of food shortage at any time in life, as indicator of poverty in general, was found associated with leprosy, while in our study only a recent period of food shortage was associated with the disease. Although a higher percentage of leprosy cases also reported food shortage at any time of life in Bangladesh, this association was not statistically significant. Different case definitions of food shortage or differences in social norms regarding nutritional requirements between the countries could be an explanation for this difference. Food shortage however, may also be a less strong indicator of poverty in general in Bangladesh than in Brazil, since the percentage of people who reported food shortage ever was much higher in Bangladesh (66.7% of the cases and 61.8% of the controls) than in Brazil (28% of the cases and 19% of the controls).

Nutritional status is known to influence the development of other infectious diseases such as respiratory infections, infectious diarrhoea, measles and malaria. These diseases are observed more commonly in malnourished children. Malnutrition affects the immune system negatively, causing infected individuals to be more vulnerable for developing a clinically apparent infection [23]. In tuberculosis, which has similarities to leprosy since it is also caused by a mycobacterium, nutritional deficit has been identified as an important risk factor in the development of clinical symptoms of disease. This is based on historical reports of outbreaks during famines and wars, and on animal studies in which cell mediated immunity was diminished in malnourished guinea pigs. Cell mediated immunity, which is affected by both protein energy malnutrition and micronutrient deficiencies, plays an important role in host defense against tuberculosis and leprosy [24]. A recent period of food shortage as identified in our study as most important poverty-related factor associated with leprosy, very likely has reduced the cell mediated immunity of individuals incubating *Mycobacterium leprae*, causing the development of clinical leprosy disease.

Targeted nutritional support to high risk groups should therefore be included in leprosy control programmes in endemic areas to reduce risk of disease. It would be useful to give contacts of leprosy patients, who are at high risk of developing leprosy themselves, dietary advices to prevent malnutrition. Because food shortage is seasonal and poverty related in northwest Bangladesh, extra attention and support should be given to the poorest families with leprosy patients. It is important to prevent malnutrition in these families to prevent clinical leprosy among contacts of patients.

## Supporting Information

Checklist S1 STROBE checklist
(PDF)
